# The role of SIRT1 level and *SIRT1* gene polymorphisms in optic neuritis patients with multiple sclerosis

**DOI:** 10.1186/s13023-023-02665-x

**Published:** 2023-03-22

**Authors:** Aleksandra Kubiliute, Greta Gedvilaite, Alvita Vilkeviciute, Loresa Kriauciuniene, Akvile Bruzaite, Dalia Zaliuniene, Rasa Liutkeviciene

**Affiliations:** 1 Medical Faculty, Lithuanian University of Health Sciences, Medical Academy, Eiveniu Str. 2, 50161 Kaunas, Lithuania; 2 Laboratory of Ophthalmology, Neuroscience Institute, Lithuanian University of Health Sciences, Medical Academy, Eiveniu Str. 2, 50161 Kaunas, Lithuania; 3 Department of Ophthalmology, Lithuanian University of Health Sciences, Medical Academy, Eiveniu 2 Str, 50161 Kaunas, Lithuania

**Keywords:** Optic neuritis, Multiple sclerosis, *SIRT1* SNP, SIRT1 ELISA

## Abstract

**The aim:**

To investigate the role of Sirtuin 1 (SIRT1) level and *SIRT1* (rs3818292, rs3758391, rs7895833) gene polymorphisms in patients with optic neuritis (ON) and multiple sclerosis (MS).

**Methods:**

79 patients with ON and 225 healthy subjects were included in the study. ON patients were divided into 2 subgroups: patients with MS (n = 30) and patients without MS (n = 43). 6 ON patients did not have sufficient data for MS diagnosis and were excluded from the subgroup analysis. DNA was extracted from peripheral blood leukocytes and genotyped by real-time polymerase chain reaction. Results were analysed using the program "IBM SPSS Statistics 27.0".

**Results:**

We discovered that *SIRT1* rs3758391 was associated with a twofold increased odds of developing ON under the codominant (p = 0.007), dominant (p = 0.011), and over-dominant (p = 0.008) models. Also, it was associated with a threefold increased odds ofON with MS development under the dominant (p = 0.010), twofold increased odds under the over-dominant (p = 0.032) models and a 1.2-fold increased odds of ON with MS development (p = 0.015) under the additive model. We also discovered that the *SIRT1* rs7895833 was significantly associated with a 2.5-fold increased odds of ON development under the codominant (p = 0.001), dominant (p = 0.006), and over-dominant (p < 0.001) models, and a fourfold increased odds of ON with MS development under the codominant (p < 0.001), dominant (p = 0.001), over-dominant (p < 0.001) models and with a twofold increased odds of ON with MS development (p = 0.013) under the additive genetic model. There was no association between SIRT1 levels and ON with/without MS development.

**Conclusions:**

*SIRT1* rs3758391 and rs7895833 polymorphisms are associated with ON and ON with MS development.

**Supplementary Information:**

The online version contains supplementary material available at 10.1186/s13023-023-02665-x.

## Introduction

Optic neuritis (ON)—inflammation of the optic nerve characterized by painful, usually monocular, visual disturbances, visual field defects, and loss of color contrast sensitivity in young people (mostly women), usually associated with multiple sclerosis (MS) [1]. MS is characterized by central nervous system lesions that cause neurologic dysfunction and other complaints such as fatigue, pain, depression, and anxiety. The disease usually relapses in the early stages, but most people develop the secondary progressive disease over time. Treatment has not been shown to affect long-term outcomes [2]. An association between ON and MS has been known for many years. In 15–20% of patients with MS, optic neuritis is the first inflammatory event, and half of MS patients have had at least one ON attack within the past 15 years [3]. Like ON, MS is a multifactorial disease with many genes and environmental factors involved in its pathogenesis.

Sirtuin 1 (SIRT1) is known to be expressed in the cornea, lens, iris, ciliary body, inner nuclear layer, outer nuclear layer, and retinal ganglion cell layer of mice [4]. It may impact on the development of ON and other neurodegenerative diseases in many experimental models [5–15]. Devin S. McDougald et al. demonstrated a significant role of the SIRT1 gene in the pathogenesis of ON and MS in experimental models [5]. SIRT1 is an evolutionarily conserved NAD + -dependent deacetylase that regulates various components of cellular metabolism related to aging, DNA repair, mitochondrial biogenesis, and apoptosis [6]. There is growing evidence that modulation of SIRT1 activity by pharmacological induction or transgenic overexpression may be of therapeutic value in various forms of neurodegenerative diseases [7]. SIRT1 mediates neuroprotection from mutant huntingtin by activating the TORC1, which is a highly conserved protein kinase that couples changes in amino acids and glucose levels with transcriptional metabolic reprogramming via its downstream effectors, and CREB—the cAMP-response element binding protein, which is an intracellular protein that regulates the expression of genes that are important in dopaminergic neurons, transcriptional pathways [8]. SIRT1-activating compounds reduce oxidative stress-mediated neuronal loss in virus-induced CNS demyelinating diseases [9–15]. In experimental optic neuritis, small-molecule activators of SIRT1, including resveratrol and related polyphenolic compounds, effectively preserve visual acuity and retinal ganglion cells (RGC) survival in EAE and virus-related demyelinating diseases [7–9]. The results suggest that SIRT1-activating drugs may play a specific role in preventing traumatic optic nerve damage and suggest a broader role for this strategy in treating various optic nerve diseases that may contain an oxidative stress component [15].

Therefore, our study aimed to investigate the role of SIRT1 levels and *SIRT1* (rs3818292, rs3758391, rs7895833*)* gene polymorphisms in patients with optic neuritis and multiple sclerosis in the Caucasian population.

## Patients and methods

Kaunas Regional Biomedical Research Ethics Committee approved the study (No. BE-2-13). The study was conducted in the Department of Ophthalmology of Lithuanian University of Health Sciences Hospital and the Neuroscience Institute of the Lithuanian University of Health Sciences. The study participants consisted of 79 subjects diagnosed with optic neuritis and 225 subjects from the control group.

The inclusion criteria for subjects with optic neuritis were described in our previous study [16].

### The diagnostic criteria of ON and MS and the exclusion criteria

Patients were excluded if they had other systemic illnesses (diabetes mellitus, oncological diseases, systemic tissue disorders, chronic infectious diseases, autoimmune diseases, conditions after organ or tissue transplantation), obscuration of the eye optic system, or because of poor fundus photography quality. Diagnosis of MS was confirmed with 2017 diagnostic criteria: clinical symptoms/relapse, brain/spinal cord MRI (Magnetic Resonance Imaging) findings with typical demyelinating lesions (according to MAGNIMS criteria), and positive oligoclonal bands.

### Sample preparation, DNA extraction, genotyping, and enzyme immunoassay

Blood samples were collected in vacutainers with EDTA. EDTA acts as an anticoagulant, binding the calcium ions and interrupting the clotting of the blood sample.

After whole blood collection, we allowed the blood to clot by leaving it undisturbed at room temperature for 30 min. The clot was removed by centrifuging at 3,000 × g for 10 min in a refrigerated centrifuge. The resulting supernatant—blood serum was stored in a fridge at -20 degrees C temperature.

We extracted DNA samples from peripheral venous blood using the DNA salting-out method. Genotyping of all three SNPs was performed using TaqMan® genotyping assays (Applied Biosystems Foster City, CA, USA): *SIRT1* (rs3818292, rs3758391, and rs7895833) according to the manufacturer's instructions using real-time polymerase chain reaction (PCR). Serum SIRT1 levels were determined in control subjects and patients using the commercial enzyme-linked immunosorbent assay (ELISA) kit for human SIRT1 (Human SIRT1 ELISA Kit, Abcam, Cambridge, United Kingdom) according to the manufacturer's instructions, and optical density was measured immediately at a wavelength of 450 nm using a microplate reader (Multiskan FC microplate photometer, Thermo Scientific, Waltham, MA). The SIRT1 level was calculated using the standard curve; the sensitivity range of the standard curve: 0.63–40 ng/ml, sensitivity 132 pg/ml.

### Quality control of genotyping

Repeated analysis of 5% randomly selected samples was performed for all SNPs to confirm the same rate of genotypes from initial and repeated genotyping.

### Statistical analysis

Statistical analysis was performed with SPSS/W 27.0 software (Statistical Package for the Social Sciences for Windows, Inc, Chicago, Illinois, USA). Data on subjects' ages were expressed as mean with standard deviation (SD) and median with interquartile range (IQR). The Student t-test was performed to compare the mean age of the study groups, and the Mann–Whitney U test was used to compare serum SIRT1 levels between the study groups. Hardy–Weinberg equilibrium analysis compared the observed and expected frequencies of *SIRT1* rs3818292, rs3758391, and rs7895833. The distributions of the genotypes and alleles in the study groups and subgroups were compared using the χ^2^ test. Binomial logistic regression analysis was performed to estimate the effects of genotypes on the development of ON, and ON subgroups: with MS and without MS. Odds ratios and 95% confidence intervals are shown. The best genetic model selection was based on the Akaike Information Criterion (AIC); therefore, the best genetic models were those with the lowest AIC values. Differences were considered statistically significant when p < 0.05.

## Results

Our study population included data from 304 individuals. To investigate the frequency of selected gene polymorphisms, subjects were divided into two groups. The group of ON patients included 79 subjects: 26 (32.9%) males and 53 (67.1%) females. The control group included 225 subjects: 91 (40.4%) males and 134 (59.6%) females. There was no statistically significant difference between males and females with ON and the control groups (p = 0.236). The mean age was 37 years in the patients with ON and 32 years in the control group. No statistically significant differences were found between the groups by age (p = 0.066). The distribution of subjects by gender and age is shown in Additional file 1: Table S1.

### Associations between *SIRT1* concentration, optic neuritis, and multiple sclerosis

Blood serum SIRT1 concentrations were determined in patients with ON (n = 23) and the control group (n = 24). Statistically significant differences were not observed between these groups (IQR: 2.130 ng/ml (1.68) vs. 2.130 ng/ml (0.61), respectively, p = 0.856).

Also, serum SIRT1 levels were compared between ON patients with MS and ON patients without MS subgroups. We also found no statistically significant differences between these two groups (IQR: 3.821 ng/ml (4.35) vs. 2.124 ng/ml (0.61), p = 0.593).

No statistically significant differences in serum SIRT1 levels were found between ON patients with MS and the control group (IQR: 3.821 ng/ml (4.35) vs. 2.130 ng/ml (0.61), p = 0.695) or between ON patients without MS and the control group (IQR: 2.124 ng/ml (0.61), 2.130 ng/ml (0.61), p = 0.989).

### *SIRT1* rs3818292, rs3758391, rs7895833 genotypes associations with ON

We determined the frequency of genotypes and alleles of *SIRT1* rs3818292, rs3758391, and rs7895833 SNPs compared between ON patients and control groups. The distribution of genotypes and alleles in the control group was in accordance with the Hardy–Weinberg equilibrium (p > 0.001). There was no statistically significant difference in the frequency of genotypes and alleles of *SIRT1* rs3818292 in ON patients and control groups (p = 0.067 and p = 0.383, respectively) (Table 1) (Table 2).

**Table 1:** *SIRT1* rs3818292, rs3758391, rs7895833 genotypes and alleles frequencies

Polymorphism	Genotype/allele	Frequency (%)			
		Control group, n (%) N = 225	HWE p-value	Patients with ON, n (%) N = 79	p-value
rs3818292	Genotype				
	A/A	192 (85.3)	0.003	62 (78.5)	0.067
	A/G	28 (12.4)		17 (21.5)	
	G/G	5 (2.2)		0 (0)	
	Total	225 (100)		79 (100)	
	Allele				
	A	412 (91.6)		141 (89.2)	0.383
	G	38 (8.4)		17 (10.8)	
rs3758391	Genotype				
	C/C	126 (56.0)^**1**^	0.334	31 (39.2)^**1**^	**0.024**
	C/T	81 (36.0)^**2**^		42 (53.2)^**2**^	
	T/T	18 (8.0)		6 (7.6)	
	Total	225 (100)		79 (100)	
	Allele				
	C	333 (74.0)		104 (65.8)	0.062
	T	117 (26.0)		54 (34.2)	
rs7895833	Genotype				
	A/A	166 (73.8)^**3**^	0.001	45 (57.0)^**3**^	**0.001**
	A/G	47 (20.9)^**4**^		33 (41.8)^**4**^	
	G/G	12 (5.3)		1 (1.3)	
	Total	225 (100)		79 (100)	
	Allele				
	A	379 (84.2)		123 (77.8)	0.069
	G	71 (15.8)		35 (22.2)	

ON—optic neuritis; p value—significance level (statistically significant when p < 0.05 (in bold)); HVE p-value—significance level according to Hardy–Weinberg equilibrium principle (differences are considered significant, when p < 0.001); 1—p = 0.010; 2—p = 0.007; 3—p = 0.007; 4—p = 0.001

**Table 2:** *SIRT1* rs3818292, rs3758391, rs7895833 binary logistic regression between ON and control groups

Polymorphism	Model	Genotype	OR (95% CI)	p-value	AIC
rs3818292	Codominant	A/A	1	0.064 1	345.990
		A/G	1.880 (0.865–3.664)		
		G/G	–		
	Dominant	A/A	1	0.160	348.426
		A/G + G/G	1.595 (0.832–3.060)		
	Recessive	AA + A/G	1	1	347.296
		G/G	–		
	Overdominant	A/A + G/G	1	0.053	346.261
		A/G	1.929 (0.990–3.758)		
	Additive	G	1.275 (0.718–2.264)	0.407	349.667
rs3758391	Codominant	C/C	1	**0.007** 0.553	344.935
		C/T	2.108 (1.226–3.622)		
		T/T	1.355 (0.496–3.698)		
	Dominant	C/C	1	**0.011**	343.729
		C/T + T/T	1.971 (1.168–3.324)		
	Recessive	C/C + C/T	1	0.909	350.322
		T/T	0.945 (0.361–2.473)		
	Overdominant	C/C + T/T	1	**0.008**	343.274
		C/T	2.018 (1.201–3.391)		
	Additive	T	1.482 (0.999–2.199)	0.051	346.545
rs7895833	Codominant	A/A	1	**0.001** 0.574	338.196
		A/G	2.590 (1.489–4.506)		
		G/G	0.574 (0.025–7.691)		
	Dominant	A/A	1	**0.006**	342.826
		A/G + G/G	2.126 (1.245–3.631)		
	Recessive	AA + A/G	1	0.158	347.368
		G/G	0.228 (0.029–1.779)		
	Overdominant	A/A + G/G	1	** < 0.001**	337.908
		A/G	2.717 (1.566–4.712)		
	Additive	G	1.470 (0.950–2.276)	0.084	347.415

p-value—significance level (differences are considered significant when p < 0.05 (in bold)); OR—odds ratio; CI—confidence interval; AIC—Akaike information criterion

There was a statistically significant difference in C/C and C/T genotypes distribution between patients with ON and control groups. The C/C genotype of *SIRT1* rs3758391 was less frequent, and the C/T genotype was more frequent in the patients of ON than in the control group (39.2% vs. 56.0%, p = 0.010; 53.2% vs. 36.0%, p = 0.007, respectively) (Table 2).

In addition, a statistically significant difference was found between the distribution of *SIRT1* rs7895833 A/A and A/G genotypes. In patients with ON, the A/A genotype was less frequent, and the A/G genotype was more frequent than in the control group (57.0% vs. 73.8%, p = 0.007; 41.8% vs. 20.9%, p = 0.001, respectively) (Table 1).

Binary logistic regression analysis of *SIRT1* rs3818292, rs3758391, and rs7895833 was performed. It was found that *SIRT1* rs3758391 C/T genotype was associated with a 2.1-fold increased probability of developing ON compared to C/C genotype (OR = 2.108; 95%. CI 1.226—3.622; p = 0.007). The C/T + T/T genotypes were associated with a twofold increased odds of developing ON compared to the C/C genotype (OR = 1.971; 95%. CI 1.168—3.324; p = 0.011) and the C/T genotype was associated with a 2.7-fold increased odds of developing ON compared to the C/C + T/T genotype (OR = 2.717; 95%. CI 1.566—4.712; p = 0.008) (Table 2).

The *SIRT1* rs7895833 A/G genotype was associated with a 2.6-fold increased likelihood of developing ON than the A/A genotype (OR = 2.590; 95% CI 1.489—4.506; p = 0.001). The A/G + G/G genotypes were associated with a 2.1-fold increased odds of developing ON compared to the A/A genotype (OR = 2.126; 95%, CI 1.245—3.631; p = 0.006), and the A/G genotype was associated with a 2.7-fold increased odds of developing ON compared to the A/A + G/G genotype (OR = 2.717; 95%, CI 1.566—4.712; p < 0.001) (Table 2).

### *SIRT1* rs3818292, rs3758391, rs7895833 genotypes associations with ON according to gender and morbidity of MS

We compared the distribution of genotypes and alleles of *SIRT1* rs3818292, rs3758391, and rs7895833 in ON and control groups by sex.

The analysis showed that *SIRT1* rs7895833 A/A genotype was less frequent, while A/G genotype was more frequent in women with ON than in the control group (56.6% vs. 74.6%, p = 0.022; 41.5% vs. 20.9%, p = 0.006, respectively. We found no statistically significant differences in the male groups (Table 3).

**Table 3:** *SIRT1* rs3818292, rs3758391, rs7895833 genotypes and alleles frequencies in patients with ON by gender

Polymorphism	Genotype	Frequency, n (%)					
		Males		p-value	Females		p-value
		Control group, n (%) N = 91	Patients with ON, n (%) N = 26		Control group, n (%) N = 134	Patients with ON, n (%) N = 53	
rs3818292	A/A	80 (87.9)	21 (80.8)	0.788	112 (83.6)	41 (77.4)	0.492
	A/G	7 (7.7)	5 (19.2)		21 (15.7)	12 (22.6)	
	G/G	4 (4.4)	0 (0)		1 (0.7)	0 (0)	
	Allele						
	A	167 (91.8)	47 (90.4)	0.755	245 (91.4)	94 (88.7)	0.412
	G	15 (8.2)	5 (9.6)		23 (8.6)	12 (11.3)	
rs3758391	Genotype						
	C/C	48 (52.7)	10 (38.5)	0.385	78 (58.2)	21 (39.6)	0.056
	C/T	32 (35.2)	13 (50)		49 (36.6)	29 (54.7)	
	T/T	11 (12.1)	3 (11.5)		7 (5.2)	3 (5.7)	
	Allele						
	C	128 (70.3)	33 (63.5)	0.346	205 (76.5)	71 (67.0)	0.594
	T	54 (29.7)	19 (36.5)		63 (23.5)	35 (33.0)	
rs7895833	Genotype						
	A/A	66 (72.5)	15 (57.7)	0.067	100 (74.6)^1^	30 (56.6)^1^	**0.014**
	A/G	19 (20.9)	11 (42.3)		28 (20.9)^2^	22 (41.5)^2^	
	G/G	6 (6.6)	0 (0)		6 (4.5)	1 (1.9)	
	Allele						
	A	151 (83.0)	41 (78.8)	0.495	228 (85.1)	82 (77.4)	0.074
	G	31 (17.0)	11 (21.2)		40 (14.9)	24 (22.6)	

ON—optic neuritis; p-value—significance level (differences are considered significant when p < 0.05, (in bold)); 1—(A/A vs. A/G + G/G) p = 0.022; 2—(A/G vs. A/A + G/G) p = 0.006

Binary logistic regression analysis of *SIRT1* rs3818292, rs3758391, and rs7895833 in patients with ON and control groups by sex was performed. In the male group, we observed that the A/G genotype of *SIRT1* rs7895833 was associated with a 2.6-fold increased likelihood of developing ON compared to the A/A genotype (OR = 2.547; 95%. CI 1.005—6.459; p = 0.049) and the A/G genotype was associated with a 2.8-fold increased odds of developing ON in males compared to the A/A and G/G genotypes (OR = 2.779; 95%. CI 1.099—7.028; p = 0.031) (Table 4).

**Table 4:** Binary logistic regression for *SIRT1* rs3818292, rs3758391, between males of ON and control groups

Polymorphism	Model	Genotype	OR (95%. CI)	p-value	AIC
rs3818292	Codominant	A/A	1	0.115 1	123.561
		A/G	2.721 (0.784–9.443)		
		G/G	–		
	Dominant	A/A	1	0.354	125.135
		A/G + G/G	1.732 (0.542–5.531)		
	Recessive	AA + A/G	1	1	123.901
		G/G	–		
	Overdominant	A/A + G/G	1	0.098	123.358
		A/G	2.857 (0.824–9.906)		
	Additive	G	1.134 (0.455–2.825)	0.788	125.881
rs3758391	Codominant	C/C	1	0.163 0.715	125.976
		C/T	1.950 (0.763–4.982)		
		T/T	1.309 (0.308–5.564)		
	Dominant	C/C	1	0.202	124.228
		C/T + T/T	1.786 (0.733–4.353)		
	Recessive	C/C + C/T	1	0.939	125.945
		T/T	0.949 (0.244–3.689)		
	Overdominant	C/C + T/T	1	0.173	124.106
		C/T	1.844 (0.764–4.450)		
	Additive	T	1.326 (0.714–2.460)	0.371	125.162
rs7895833	Codominant	A/A	1	**0.049** 1	121.054
		A/G	2.547 (1.005–6.459)		
		G/G	–		
	Dominant	A/A	1	0.152	123.941
		A/G + G/G	1.936 (0.784–4.781)		
	Recessive	AA + A/G	1	1	122.844
		G/G	–		
	Overdominant	A/A + G/G	1	**0.031**	121.416
		A/G	2.779 (1.099–7.028)		
	Additive	G	1.267 (0.614–2.611)	0.522	125.551

p-value—significance level (differences are considered significant when p < 0,05 (in bold)); OR—odds ratio; CI—confidence interval; AIC—Akaike information criterion

In the female group, we found that the C/T genotype of *SIRT1* rs3758391 was associated with a 2.2-fold increased likelihood of developing ON compared with the C/C genotype (OR = 2.198; 95%. CI 1.130—4.277; p = 0.020). The C/T + T/T genotypes were associated with a 2.1-fold increased odds of developing ON compared to the C/C genotype (OR = 2.122; 95%. CI 1.109—4.060; p = 0.023) and the C/T genotype was also associated with a 2.1-fold increased odds of developing ON compared to the C/C and T/T genotypes in females (OR = 2.096; 95%. CI 1.100—3.995; p = 0.025) (Table 6). The A/G genotype of *SIRT1* rs7895833 was associated with a 2.6-fold increased probability of developing ON compared to the A/A genotype (OR = 2.619; 95%. CI 1.312—5.230; p = 0.006). Genotypes A/G + G/G were associated with a 2.2-fold increased odds of developing ON compared to genotype A/A (OR = 2.255; 95%. CI 1.156—4.399; p = 0.017), and genotype A/G was associated with a 2.7-fold increased odds of developing ON in females compared to genotype A/A + G/G (OR = 2.68; 95%. CI (1.352—5.340); p = 0.005) (Table 5).

**Table 5:** Binary logistic regression for *SIRT1* rs3818292, rs3758391, between females of ON and control groups

Polymorphism	Model	Genotype	OR (95%. CI)	p-value	AIC
rs3818292	Codominant	A/A	1	0.272 1	225.119
		A/G	1.561 (0.705–3.455)		
		G/G	–		
	Dominant	A/A	1	0.322	224.006
		A/G + G/G	1.490 (0.677–3.280)		
	Recessive	AA + A/G	1	1	224.294
		G/G	–		
	Overdominant	A/A + G/G	1	0.262	223.741
		A/G	1.575 (0.712–3.485)		
	Additive	G	1.379 (0.648–2.937)	0.405	224.285
rs3758391	Codominant	C/C	1	**0.020** 0.526	221.480
		C/T	2.198 (1.130–4.277)		
		T/T	1.592 (0.379–6.690)		
	Dominant	C/C	1	**0.023**	219.682
		C/T + T/T	2.122 (1.109–4.060)		
	Recessive	C/C + C/T	1	0.905	224.948
		T/T	1.089 (0.271–4.377)		
	Overdominant	C/C + T/T	1	**0.025**	219.862
		C/T	2.096 (1.100–3.995)		
	Additive	T	1.684 (0.995–2.849)	0.052	221.181
rs7895833	Codominant	A/A	1	**0.006** 0.593	218.788
		A/G	2.619 (1.312–5.230)		
		G/G	0.556 (0.064–4.798)		
	Dominant	A/A	1	**0.017**	219.336
		A/G + G/G	2.255 (1.156–4.399)		
	Recessive	AA + A/G	1	0.415	224.157
		G/G	0.410 (0.048–3.492)		
	Overdominant	A/A + G/G	1	**0.005**	217.111
		A/G	2.687 (1.352–5.340)		
	Additive	G	1.627 (0.933–2.837)	0.086	222.068

p-value—significance level (differences are considered significant when p < 0,05 (in bold)); OR—odds ratio; CI—confidence interval; AIC—Akaike information criterion

We studied the genotypes and allele distribution of *SIRT1* rs3818292, rs3758391, and rs7895833 in ON patients and control groups with and without MS.

We found that the *SIRT1* rs3758391 C/C was less frequent, and the C/T genotype was more frequent in ON patients with MS than in the control group (30%. vs. 60%, p = 0.007; 56.7%. vs. 36%, p = 0.029, respectively). The T allele was more frequent in ON patients with MS compared to the control group (41.7% vs. 26.0%, p = 0.011) (Table 6).

**Table 6:** *SIRT1* rs3818292, rs3758391, rs7895833 genotypes and alleles frequencies for ON patients with and without MS and control group

Polymorphism	Genotype	Frequencies, n (proc.)				
		Control group, n (%) N = 225	ON patients with MS, n (%) N = 30	p-value	ON patients with MS, n (%) N = 43	p-value
rs3818292	Genotype					
	A/A	192 (85.3)	23 (76.7)	0.243	34 (79.1)	0.250
	A/G	28 (12.4)	7 (23.3)		9 (20.9)	
	G/G	5 (2.2)	0 (0)		0 (0)	
	Allele					
	A	412 (91.6)	53 (88.3)	0.408	77 (89.5)	0.544
	G	38 (8.4)	7 (11.7)		9 (10.5)	
rs3758391	Genotype					
	C/C	126 (56.0)^1^	9 (30.0)^1^	**0.023**	20 (46.5)	0.270
	C/T	81 (36.0)^2^	17 (56.7)^2^		21 (48.8)	
	T/T	18 (8.0)	4 (13.3)		2 (4.7)	
	Allele					
	A	333 (74.0)	35 (58.3)	**0.011**	61 (70.9)	0.554
	G	117 (26.0)	25 (41.7)		25 (29.1)	
rs7895833	Genotype					
	A/A	166 (73.8)^3^	13 (43.4)^3^	**0.001**	29 (67.4)	0.107
	A/G	47 (20.9)^4^	16 (53.3)^4^		14 (32.6)	
	G/G	12 (5.3)	1 (3.3)		0 (0)	
	Allele					
	A	379 (84,2)	42 (70,0)	**0.006**	72 (83.7)	0.907
	G	71 (15,8)	18 (30,0)		14 (16.3)	

ON—optical neuritis; MS—multiple sclerosis; p-value—significance level (differences are considered significant, when p < 0,05 (in bold)); 1—p = 0,007; 2—p = 0,029; 3—p = 0,001; 4—p < 0,001

In addition, statistically significant differences were found in the distribution of *SIRT1* rs895833 genotypes A/A, A/G, and G/G in ON patients with MS and the control group (p = 0.001). The A/A genotype was less frequent, and the A/G genotype was more frequent in ON patients with MS than in the control group (43.4% vs. 73.8%, p = 0001; 53.3% vs. 20.9%, p < 0.001, respectively). The G allele was also more frequent in ON patients with MS than in the control group (30.0% vs. 15.8%, p = 0.006) (Table 6).

We performed binary logistic regression analysis to evaluate the influence of *SIRT1* rs3818292, rs3758391, and rs7895833 on the development of ON in patients with and without MS.

We found that the C/T and T/T genotypes *SIRT1* rs3758391 together were associated with a threefold increased odds of ON patients with MS (OR = 2.970; 95%. CI 1.303—6.770; p = 0.010), and the C/T genotype was associated with a 2.2-fold increased odds of ON patients with MS while compared with C/C and T/T genotypes (OR = 2.235; 95% CI 1.075—5.030; p = 0.032). The T allele was associated with a 1.2-fold increased odds of ON with MS (OR = 1.199; 95% CI 1.143—3.450; p = 0.015) (Table 7).

**Table 7:** Binary logistic regression for *SIRT1* rs3818292, rs3758391, between ON patients with MS and the control group

Polymorphism	Model	Genotype	OR (95% CI)	p-value	AIC
rs3818292	Codominant	A/A	1	0.123 1	185,292
		A/G	2.087 (0.820–5.312)		
		G/G	–		
	Dominant	A/A	1	0.225	185.362
		A/G + G/G	1.771 (0.703–4.457)		
	Recessive	AA + A/G	1	1	185.462
		G/G	–		
	Overdominant	A/A + G/G	1	0.110	184.409
		A/G	2.141 (0.841–5449)		
	Additive	G	1.364 (0617–3.013)	0.443	186.178
rs3758391	Codominant	C/C	1	0.082 0.926	181.417
		C/T	0.321 (0.090–1.153)		
		T/T	0.994 (0.284–3.145)		
	Dominant	C/C	1	**0.010**	179.425
		C/T + T/T	2.970 (1,303–6.770)		
	Recessive	C/C + C/T	1	0.334	185.879
		T/T	1.769 (0.556–5.630)		
	Overdominant	C/C + T/T	1	**0.032**	182.090
		C/T	2.235 (1.075–5.030)		
	Additive	T	1.199 (1.143–3450)	**0.015**	178.919
rs7895833	Codominant	A/A	1	** < 0.001** 0.954	175.665
		A/G	4.347 (1.953–9.677)		
		G/G	1.064 (0.128–8.836)		
	Dominant	A/A	1	**0.001**	176.008
		A/G + G/G	3.679 (1.685–8.033)		
	Recessive	AA + A/G	1	0.643	186.483
		G/G	0.612 (0.077–4.882)		
	Overdominant	A/A + G/G	1	** < 0.001**	173.668
		A/G	4.328 (1.972–9.499)		
	Additive	G	2.058 (1.162–3.645)	**0.013**	181.025

p-value—significance level (differences are considered significant when p < 0,05 (in bold)); OR—odds ratio; CI—confidence interval; AIC—Akaike information criterion

The A/G genotype of *SIRT1* rs7895833 was associated with a 4.4-fold increased odds of developing ON with MS compared with the A/A genotype (OR = 4.347; 95%. CI 1.953—9.677; p < 0.001), A/G + G/G genotypes were associated with 3.7-fold increased odds of ON in patients with MS compared to A/A genotype (OR = 3.679; 95% CI 1.685—8.033; p = 0.001) and A/G genotype was associated with 4.3-fold increased odds of ON in patients with MS compared to A/A and G/G genotype (OR = 4.328; 95% CI 1.972—9.499; p < 0.001). At least one G allele was associated with a 2.1-fold increased odds of ON with MS (OR = 2.058; 95% CI 1.162–3.645; p = 0.013) (Table 7). Unfortunately, no statistically significant results were found while ON patients without MS, and the control group were analysed ( Additional file 1: Table S1).

### *SIRT1* serum levels and *SIRT1* rs3818292, rs3758391, rs7895833 associations with ON

We compared the serum levels of ON patients and the control group according to the *SIRT1* SNPs genotypes. Because of the small subject group, we formed two groups: homozygous with the more common allele and heterozygous and homozygous with the less common allele together.

First, serum SIRT1 levels were compared in all subjects. There was no statistically significant difference in SIRT1 serum levels between subjects with the A/A genotype and with the A/G and G/G genotypes for the *SIRT1* rs3818292 (IQR: 2.130 ng/ml (0.54) vs. 2.124 ng/ml (1.68), p = 0.551). Similarly, serum SIRT1 levels did not differ between *SIRT1* rs3758391 C/C genotype and C/T + T/T genotypes (IQR: 2.130 ng/ml (0.54) vs. 1.124 ng/ml (1.53), p = 1). No statistically significant difference was found between SIRT1 rs7895833 polymorphism A/A genotype and A/G + G/G genotypes (IQR: 2.130 ng/ml (0.51) vs. 2.130 ng/ml (1.57), p = 0.915).

No statistically significant difference in SIRT1 serum levels was found between subjects with A/A genotype and with A/G and G/G genotypes for *SIRT1* rs3818292 (IQR: 2.174 ng/ml (1.51) vs. 1.911 ng/ml (1.85), respectively, p = 0.379). Serum SIRT1 levels did not differ between *SIRT1* rs3758391 C/C genotype and C/T and T/T genotypes (IQR: 2.130 ng/ml (1.85) vs. 2.134 ng/ml (1.77), p = 0.525). In addition, there was no statistically significant difference in SIRT1 serum levels between the *SIRT1* rs7895833 A/A genotype and the A/G + G/G genotypes (IQR: 2.130 ng/ml (1.85) vs. 2.134 ng/ml (1.77), p = 0.525.

No statistically significant differences in serum SIRT1 levels were found between subjects with A/A genotype and with A/G + G/G genotypes for the *SIRT1* rs3818292 (IQR: 2.130 ng/ml (0.58) vs. 2.130 ng/ml (1.65), respectively, p = 0.910). Serum SIRT1 levels did not differ between SIRT1 rs3758391 C/C genotype and C/T and T/T genotypes (IQR: 2.066 ng/ml (0.55) vs. 2.188 ng/ml (1.26), respectively, p = 0.472). In addition, there was no statistically significant difference in SIRT1 serum levels between the *SIRT1* rs7895833 A/A genotype and the A/G + G/G genotypes (IQR: 2.130 ng/ml (0.52) vs. 2.130 ng/ml (1.57), p = 0.424).

## Discussion

Optic neuritis is an inflammatory optic neuropathy often associated with multiple sclerosis [17, 18]. Typically, myelin ensures that electrical impulses travel rapidly from the eye to the brain, converting them into visual information. Optic neuritis disrupts this process and impairs vision [19]. It is well known that genetic factors may influence ON and MS pathogenesis. Therefore, our study investigated the role of SIRT1 level and *SIRT1* (rs3818292, rs3758391, rs7895833) gene polymorphisms in patients with optic neuritis and multiple sclerosis.

There is growing interest that modulation of SIRT1 activity by pharmacological induction or transgenic overexpression may be of therapeutic value in various neurodegenerative diseases. These experimental models are discussed in the following discussion [5, 7, 10, 14, 15, 20–22].

SIRT1 is one of the targets of resveratrol, which has been shown to increase longevity and protect various organs from aging. SIRT1 is localized in the nucleus and cytoplasm of cells that form all typical ocular structures, including the cornea, lens, iris, ciliary body, and retina, and that it may provide protection against diseases related to oxidative stress-induced ocular damage [23] and also plays a role in DNA repair, mitochondrial biogenesis, and apoptosis [6].

There are many animal studies, but no studies investigating the role of SIRT1 level and *SIRT1* (rs3818292, rs3758391, rs7895833) gene polymorphisms in patients with optic neuritis and multiple sclerosis [24]. It is known that intravitreal injection of SIRT1 agonists inhibits the loss of RGCs in a dose-dependent manner by inducing SIRT1 activity in mice with optic neuritis. This neuroprotective effect is blocked by sirtinol [25].

In contrast to SIRT1 overexpression, SIRT1 inactivation in an established mouse model of multiple sclerosis increased the production of new oligodendrocyte progenitor cells in the adult mouse brain, improved remyelination, and delayed paralysis [26]. Sirtuins have received considerable attention since the discovery that Silent Information Regulator 2 (Sir2) extends yeast lifespan [24]. Sir2, a nicotinamide adenine dinucleotide (NAD -) dependent histone deacetylase, is a transcriptional effector and an energy sensor. Oxidative stress and apoptosis are associated with the pathogenesis of neurodegenerative eye diseases. Sirtuins provide protection against oxidative stress and retinal degeneration [26].

In mammals, the SIRT family consists of seven proteins. These differ in tissue specificity, subcellular localization, enzymatic activity, and targets. A possible role of specific therapeutic targets is currently being explored [27].

Khan and colleagues investigated whether SIRT1 activators reduce oxidative stress and promote mitochondrial function in neuronal cells. Furthermore, the results suggest that SIRT1 activators may mediate neuroprotective effects during optic neuritis and potentially preserve neurons in other neurodegenerative diseases associated with oxidative stress [10]. In another study, Guo J et al. note that patients with multiple sclerosis often accompany ON, leading to RGC death and even vision loss [20]. Other investigators examined the potential neuroprotective effects of SRT647 and SRT501, two structurally and mechanistically distinct activators of SIRT1, an enzyme involved in cellular stress resistance and survival in optic neuritis. They used experimental EAE, an animal model of MS, induced by immunization with proteolipid protein peptide in SJL/J mice. Optic neuritis developed in two-thirds of the eyes, with significant loss of retinal ganglion cells (RGCs) 14 days after immunization. The RGCs were retrogradely labeled with fluorogold by injection into the superior colliculi. Optic neuritis was detected by infiltration of the optic nerve with inflammatory cells. Intravitreal injection of SIRT1 activators 0, 3, 7, and 11 days after immunization significantly reduced RGC loss in a dose-dependent manner. This neuroprotective effect was blocked by sirtinol, a SIRT1 inhibitor. Treatment with either SIRT1 activator did not prevent EAE or optic nerve inflammation. A single administration of SRT501 on day 11 was sufficient to limit RGC loss and preserve axon function [14]. Another experimental study showed that optic nerve crush was induced in wild-type C57BL/6 mice, in mice overexpressing SIRT1, and in mice with conditional deletion of SIRT1 in neurons. Wild-type mice were treated daily with vehicle or 250 mg/kg resveratrol, a naturally occurring polyphenol that activates SIRT1. RGC function was assessed by pupillometry and optokinetic response (OKR), and RGC survival was measured. Superoxide levels were measured to assess oxidative stress. This study showed that SIRT1 delayed the loss of RGCs after traumatic injury. The effects are associated with decreased oxidative stress. The results suggest that SIRT1-activating drugs may play a specific role in preventing traumatic optic nerve damage and suggest a broader role for this strategy in treating various optic nerve diseases that may involve an oxidative stress component [15].

Balaiya S and others investigated the role of SIRT1 in maintaining RGC viability in an in vitro model of hypoxia. The role of SIRT1 in promoting viability was determined indirectly via sirtinol (SIRT1 inhibitor). Hypoxia-induced apoptosis was assessed by measuring stress-activated protein kinase/c-jun N-terminal kinase (SAPK/JNK) and caspase 3 activity. The researchers demonstrated that SIRT1 significantly affected the viability of RGCs. The effect of sirtinol reflects the interaction that SIRT1 has with apoptotic signaling proteins. This study demonstrated that SIRT1 is important in preventing the effects of hypoxia-induced apoptosis [27]. Fonseca-Kelly Z et al. investigate resveratrol's potential neuroprotective and immunomodulatory effects in chronic experimental autoimmune encephalomyelitis induced by immunization with myelin oligodendroglial glycoprotein peptide in C57/Bl6 mice. The effects of two different formulations of resveratrol administered orally daily were compared. Resveratrol delayed the onset of EAE compared with vehicle-treated EAE mice but did not prevent or alter the phenotype of inflammation in the spinal cord or optic nerves. Significant neuroprotective effects were observed, with higher numbers of retinal ganglion cells found in the eyes of resveratrol-treated EAE mice with optic neuritis. The results indicate that resveratrol prevents neuronal cell loss in this chronic demyelinating disease model, similar to recurrent EAE. Differences in immunosuppression compared with previous studies suggest that immunomodulatory effects may be limited and dependent on specific immunization parameters or the timing of treatment. Importantly, neuroprotective effects may occur even without immunosuppression, suggesting a potential additional benefit of resveratrol combined with anti-inflammatory therapies for MS [7].

## Conclusions

Our study found that the *SIRT1* polymorphisms rs3758391 and rs7895833 are associated with ON and ON during MS development, in contrast to other experimental animal models showing that *SIRT1* is a potential candidate for the treatment of MS. Therefore, the investigation of these polymorphisms should be repeated in further studies to understand better their role in ON and MS and animal models.

## Supplementary Information

**Additional file 1.** Supplementary material.

## Data Availability

The data used to support the findings of this study are available from the corresponding author upon request.

## Abbreviations

AIC: Akaike Information Criterion
CNS: Central nervous system
CREB (cAMP-response element binding protein): Is an intracellular protein that regulates the expression of genes that are important in dopaminergic neurons.
DNA: Deoksiribonucleinic acid
EAE: Is a complex condition in which the interaction between a variety of immunopathological and neuropathological mechanisms leads to an approximation of the key pathological features of MS: inflammation, demyelination, axonal loss and gliosis
ELISA: Enzyme-linked immunosorbent assay
MRI: Magnetic Resonance Imaging
MS: Multiple sclerosis
OKR: Optokinetic response
ON: Optic neuritis
RGC (retinal ganglion cell): Is a type of neuron located near the inner surface (the ganglion cell layer) of the retina of the eye
SNP: Single nucleotide polymorphism
SIRT1: Sirtuin 1
TORC1: A highly conserved protein kinase that couples changes in amino acids and glucose levels with transcriptional metabolic reprogramming via its downstream effectors, namely Sch9/S6K kinase and Tap42/ PP2A and Sit4/PP6 protein phosphatases
