# Supplementary material for: The role of SIRT1 level and SIRT1 gene polymorphisms in optic neuritis patients with multiple sclerosis
Source: Orphanet J Rare Dis. 2023 Mar 22;18:64. doi: 10.1186/s13023-023-02665-x (PMC10031967; doi:10.1186/s13023-023-02665-x)
Supplement: Supplementary file 1 — Additional file 1. Supplementary material. [file 13023_2023_2665_MOESM1_ESM.docx]

Supplementary material

**Table S1.** Demographic characteristics of study subjects*.*

| Characteristic | Group | | p-value |
| --- | --- | --- | --- |
|  | Patients with ON | Reference group |  |
| Males, n (%) | 26 (32.9) | 91 (40.4) | 0.236 |
| Females, n (%.) | 53 (67.1) | 134 (59.6) |  |
| The median age (IQR) | 37 (23) | 32 (17) | 0.066 |

ON – optic neuritis; IQR – interquartile range; p-value – significance level (statistically significant, when p<0.05);

**Table S2*.*** Binary logistic regression for *SIRT1* rs3818292, rs3758391, between ON patients without MS and the control group.

| Polymorphism | Model | Genotype | OR (95% CI) | p-value | AIC |
| --- | --- | --- | --- | --- | --- |
| rs3818292 | Codominant | A/A | 1 | 0.162  1 | 236.466 |
|  |  | A/G | 1.815 (0.788 – 4.183) |  |  |
|  |  | G/G | – |  |  |
|  | Dominant | A/A | 1 | 0.303 | 237.056 |
|  |  | A/G + G/G | 1.540 (0.677 – 3.505) |  |  |
|  | Recessive | AA + A/G | 1 | 1 | 236.294 |
|  |  | G/G | – |  |  |
|  | Overdominant | A/A + G/G | 1 | 0.144 | 236.077 |
|  |  | A/G | 1.862 (0.808 – 4.291) |  |  |
|  | Additive | G | 1.230 (0.602 – 2.516) | 0.570 | 237.752 |
| rs3758391 | Codominant | C/C | 1 | 0.153  0.649 | 237.368 |
|  |  | C/T | 1.633 (0.833 – 3.201) |  |  |
|  |  | T/T | 0.700 (0.151 – 3.249) |  |  |
|  | Dominant | C/C | 1 | 0.254 | 236.755 |
|  |  | C/T + T/T | 1.464 (0.761 – 2.816) |  |  |
|  | Recessive | C/C + C/T | 1 | 0.450 | 237.405 |
|  |  | T/T | 0.561 (0.125 – 2.511) |  |  |
|  | Overdominant | C/C + T/T | 1 | 0.115 | 235.592 |
|  |  | C/T | 1.697 (0.880 – 3.273) |  |  |
|  | Additive | T | 1.162 (0.701 – 1.927) | 0.599 | 237.724 |
| rs7895833 | Codominant | A/A | 1 | 0.144  1 | 233.706 |
|  |  | A/G | 1.705 (0.834 – 3.487) |  |  |
|  |  | G/G | – |  |  |
|  | Dominant | A/A | 1 | 0.394 | 237.351 |
|  |  | A/G + G/G | 1.358 (0.672 – 2.745) |  |  |
|  | Recessive | AA + A/G | 1 | 1 | 233.757 |
|  |  | G/G | – |  |  |
|  | Overdominant | A/A + G/G | 1 | 0.098 | 233.447 |
|  |  | A/G | 1.828 (0.895 – 3.735) |  |  |
|  | Additive | G | 1.033 (0.576 – 1.853) | 0.913 | 238.049 |

p-value - significance level (differences are considered significant when p<0,05); OR – odds ratio; CI – confidence interval; AIC – Akaike information criterion;
